# The role of enriched environment in neural development and repair

**DOI:** 10.3389/fncel.2022.890666

**Published:** 2022-07-21

**Authors:** Yu Han, Mei Yuan, Yi-Sha Guo, Xin-Ya Shen, Zhen-Kun Gao, Xia Bi

**Affiliations:** ^1^Department of Sport Rehabilitation, Shanghai University of Sport, Shanghai, China; ^2^Department of Rehabilitation Medicine, Shanghai University of Medicine and Health Sciences Affiliated Zhoupu Hospital, Shanghai, China; ^3^Department of Graduate School, Shanghai University of Medicine and Health Sciences Affiliated Shanghai University of Traditional Chinese Medicine, Shanghai, China

**Keywords:** enriched environment, CNS disorders, neurodevelopment, neurorestoration, cerebral ischemia

## Abstract

In addition to genetic information, environmental factors play an important role in the structure and function of nervous system and the occurrence and development of some nervous system diseases. Enriched environment (EE) can not only promote normal neural development through enhancing neuroplasticity but also play a nerve repair role in restoring functional activities during CNS injury by morphological and cellular and molecular adaptations in the brain. Different stages of development after birth respond to the environment to varying degrees. Therefore, we systematically review the pro-developmental and anti-stress value of EE during pregnancy, pre-weaning, and “adolescence” and analyze the difference in the effects of EE and its sub-components, especially with physical exercise. In our exploration of potential mechanisms that promote neurodevelopment, we have found that not all sub-components exert maximum value throughout the developmental phase, such as animals that do not respond to physical activity before weaning, and that EE is not superior to its sub-components in all respects. EE affects the developing and adult brain, resulting in some neuroplastic changes in the microscopic and macroscopic anatomy, finally contributing to enhanced learning and memory capacity. These positive promoting influences are particularly prominent regarding neural repair after neurobiological disorders. Taking cerebral ischemia as an example, we analyzed the molecular mediators of EE promoting repair from various dimensions. We found that EE does not always lead to positive effects on nerve repair, such as infarct size. In view of the classic issues such as standardization and relativity of EE have been thoroughly discussed, we finally focus on analyzing the essentiality of the time window of EE action and clinical translation in order to devote to the future research direction of EE and rapid and reasonable clinical application.

## Introduction

Complex genetic backgrounds and epigenetic processes shape the basic structure and function of the mammalian brain at birth for normal neural development. However, in the whole life cycle, the addition of various stimulation from the environment and the interaction with genes form a precise neural circuit structure with complexity far exceeding the content of genomic information, in which highly ordered neuronal activities are the basis of advanced brain function (Sur and Leamey, 2001; Giandomenico et al., 2021). The demonstration that the adaptation of neural networks with the changing environment manifests in subtle diversity, including but not limited to neurogenesis, neurite growth, and synaptic transmission to promote the emergence of optimal phenotypes, has emphasized the importance of environmental factors on neuroplasticity (Guzzetta et al., 2019). However, the complex interaction of numerous signaling pathways and regulatory factors with environmental factors may cause maladaptive plasticity. For instance, stress in the living environment is a risk factor for development-related mental diseases and aging-related neurodegeneration, such as depression, schizophrenia, and Alzheimer’s disease (AD; Aznar and Knudsen, 2011). The environmental parameters have attracted far more attention in neurodevelopment and repairing neurological dysfunction due to the limited therapeutic effect of drugs and the relatively uncontrollable genetic background.

Animal models have been widely used to study neurodevelopment and neural restoration, as well as to explore the pathogenesis underlying central nervous system (CNS) disease. Significantly, most animals involved in the study are raised in standard laboratory cages, which are relatively sterile environments without stimulation. The experimental animals receive more physical and social stimulation than standard rearing conditions. Experimental animals can selectively conduct physical movement, exploration, cognition, and social interaction to improve learning and memory by reshaping brain neural network construction, finally presenting better behavior performance (Nithianantharajah and Hannan, 2006). Initially, the EE studies produced impressive changes in neuroanatomy and etiology. Increased cortical thickness (Bennett et al., 1969), dendrite branching, synaptic density (Altschuler, 1979), and glial cell proliferation (Altman and Das, 1964) provide robust evidence to dissect the enhanced learning and memory skills associated with EE (van Praag et al., 2000). During the past two decades, the fundamental studies of EE at behavioral, molecular, and cellular levels have indicated that EE can be regarded as a resultful rehabilitation strategy without side-effect in stroke (Yuan et al., 2021), AD (Rodríguez et al., 2013), Parkinson’s disease (PD; Goldberg et al., 2011), Huntington’s disease (HD; Couly et al., 2021), spinal cord injury (Hutson et al., 2019), and various neurological disorders.

The implementation of concrete schemes to achieve the transformation of clinical efficacy is the path for any drug or intervention to succeed. A study focused on transforming EE intervention from the laboratory to the clinical setting demonstrated the feasibility of using EE correctly with patients in a rehabilitation setting (Janssen et al., 2012). However, the richness of the environment is relative in virtue of the different social backgrounds that humans come into contact with and the plentiful uncontrollable variables in the environment. In addition, the stimulating patterns of EE are codetermined by the patient’s own choice based on interest and the existing environmental facility setting of the hospital. The relativity of environmental richness and the diversity of collocation imply that it is hard to standardize EE, which may be one of the factors contributing to the unstable clinical efficacy of EE owing to the lack of specificity in particular CNS diseases. In this review, the mechanism underlying EE in promoting neurodevelopment and neural repair following CNS disorders is probed, and the classic criticisms and future challenges facing EE.

## EE and The Rearing Condition

**Figure 1:**
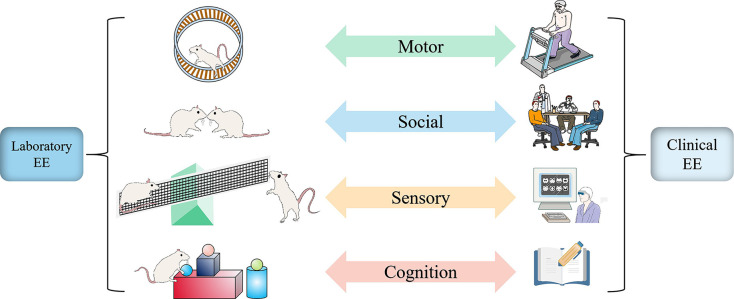
Similarity and correspondence between laboratory EE setting and clinical EE intervention. Whether laboratory or clinical, EE always provides motor, sensory, social, and cognitive stimulation to achieve positive changes in function and structure. In physical activity, animals are voluntarily active on a running wheel to match the patient on a treadmill or more diverse exercise (Green); in social stimulation, EE in the laboratory allows for extensive social interaction between animals by increasing the number of animals to match patients in the ward or treatment hall to communicate with other patients or doctors about their conditions or preferred treatment options (Blue). In sensory, animals are involved in touching a series of toys, such as seesaws, swings, and pipes, to match the patient’s experience of proprioception, touch, hearing, and other stimuli through virtual reality technology (Yellow). In terms of cognition, Animals recognize the color and shape of objects to match the patient’s participation in reading, playing games, and chess according to their interests to improve learning and memory skills (Red).

The concept of EE was first put forward by Donald Hebb, who found that rats moving freely at home possess better learning and memory capacity than those reared in the laboratory. Such an uncontrolled home environment seems different from the controllability of EE in the laboratory. Still, both reveal the critical feature of enrichment in rearing condition: ensuring the complexity and novelty of the living environment (Nithianantharajah and Hannan, 2006). Although the experimental paradigms and clinical interventions of EE present diversification, they all adopt a complex combination of non-biological and social stimuli to achieve the purpose of enriching the environment with more multi-sensory stimulation, physical activity, and social interaction (Will et al., 2004; Figure 1).

### Construction of EE in the animal model

Standard conditions for laboratory animals are free of anything other than bedding, adequate food, and water. However, besides primary living conditions, EE provides animals with more motor, sensory, cognitive, and social stimulation, which are four classical mechanisms actuating EE function (Sztainberg and Chen, 2010). Animals are born with the ability to explore. In an exploration environment with abundant and novel toys, including running wheels, tunnels, and building blocks, spontaneous somatic movements such as chasing, running, and jumping can activate motor cortex neurons (Clarke et al., 2014). Colorful, different textures of toys, some can emit music (Li et al., 2021) and unique smells, providing animals with a full range of multi-sensory stimulation, including vision, proprioception, hearing, and smell (Walsh and Cummins, 1975). In addition to ensuring the complexity of the setting in the environment, the types and locations of the built-in objects constantly change in time and space to create novelty, which improves cognition through spatial perception, object recognition, regulation of attention. Early study has observed that the high proliferation of hippocampal neurons accompanies the improvement of learning and memory ability of EE-fed mice (Kempermann et al., 1997).

Further, the EE cage has more animals and larger activity space to supply social stimulation compared with the standard cage. Properly arranged social environments provide more opportunities for information transmission and emotional exchange among animals. There are differences between social and inanimate stimuli in rearing condition, in which the social environment is relatively uncontrollable under the individual influence. Rich rearing condition provide shared living environments for animals. In contrast, some animals can change their social structure based on their behavior to create unshared environments that affect other individuals in EE cages (Kempermann, 2019). For instance, territorial male rats often fight. Therefore, even with the EE paradigm in the laboratory, environmental factors are not entirely controllable. In general, the standard for EE cage specifications has not been affirmed. However, as long as the living habits of animals are followed and the complexity and novelty of EE rearing condition are satisfied, diverse EE paradigms both produce expected positive benefits on animal behavior and brain structure.

### Construction of EE in clinical research

Hospitalized patients with CNS disorders, especially stroke patients with motor and cognitive impairments, spend most of their time with a lack of voluntary activity, if any, as part of routine rehabilitation and are highly dependent on others (Keith and Cowell, 1987; King et al., 2011). In addition, compared to the daily living environment, the ward is a barren environment, with little access to external social stimulation and social activities of inpatients with anxiety accompanied by psychological disorders, even depression (Ayerbe et al., 2013; Campbell Burton et al., 2013). Researches indicate that patients accepting higher levels of physical activity based on impaired body restriction and addition of cognitive (Shimada et al., 2018) and social activity (Lindsay et al., 2019) exert better prognosis and independence (Scrivener et al., 2012).

The forceful rationale that EE has achieved beneficial results in disease models is provided for direct or indirect translation of EE to recovery humans in clinical practice.

A study that has demonstrated 2 weeks of EE can improve activity levels in patients undergoing routine rehabilitation after stroke divided patients’ living environment into public and individual areas. Patients entering the communal environment were given access to an internet computer, various reading materials (newspaper, magazine, and fiction books), games, jigsaw puzzles, and a shared dining hall. Individual enrichment provided participants with special requirements referring to their interests and hobbies, including music, audiobooks, and number puzzles. The choice of activities and equipment was up to patients themselves, without compulsion. Nursing and rehabilitation personnel were only responsible for encouraging inactive patients to enter public areas and providing guided services to resolve operational difficulties if required (Janssen et al., 2012, 2014).

In addition to stroke, EE has also contributed significantly to promoting cognitive function in aging or dementia patients. Train the Brain Consortium has made outstanding dedications to clinical research on EE and cognitive dysfunction. In recent years, they have published clinical data associated with brain health indicators showing that comprehensive physical or cognitive training dramatically decreasing neuropsychiatric symptoms and improved the quality of life to restore cognitive status in patients with mild cognitive impairment (Train the Brain Consortium, 2017; Cintoli et al., 2021). The mechanism may be closely related to improvements in vascular function including amelioration of endothelial function throughout the body, mobilization of CD34^+^ progenitor cells, and maintenance of carotid artery dilation, especially in patients with hypertension (Bruno et al., 2018). It is well known that dysrhythmia of rest-activity is closely related to cognitive dysfunction for dementia patients (Coogan et al., 2013). One study showed that a small scaled homelike special care unit (SCU), identified as a pattern of EE by the authors, could positively affect the rest-activity rhythms of dementia patients. Compared to a regular SCU, the small scaled homelike SCU provided patients with separate bedrooms and psychosocial treatment, including stimulation of social activities and communication technology, which supplies a more silent environment and alleviates the psychological burden for better rest (Kok et al., 2017). Recent research has really elevated EE to the level of transforming the natural environment in which humans live. The enrichment garden (EG) with 12 stimulation modules, derived from an exceptional renovation of the traditional sensory garden, offers a new treatment for dementia patients in nursing homes. Patients accompanied by caretakers and staff, walking in EG for 10 to 20 min per day, and the path across the EG and location of the stimulus module they tarry can be different each time. For example, the module of sensory amplification achieves the purpose of sensitive stimulation by feeling the sensory gradation of this helicoidal pyramid and possibly modifying planting (Bourdon and Belmin, 2021). Of course, scholars attracted by EE are not limited to research CNS diseases in the elderly. A systematic review hinted that EE seems to be promising for exercise outcomes in cerebral palsy infants (Morgan et al., 2013). EE intervention for infants with cerebral palsy is usually a combination of the following aspects: parent-child interaction with the participation of parents; modification, adjustment, or construction of the treatment room or family environment, including the size, form, placement, and color of facilities to achieve motor skill acquisition; targeted motor skill and cognitive improvement exercises provided by therapists.

Obviously, the clinical application of EE has been carried out in different diseases. Drawing on the construction of experimental EE, clinical EE studies also reflect the combination of sensory stimulation, cognitive training, social activities and exercise. Therefore, it is imperative that a larger sample size and stricter environmental control be put into clinical research on EE.

## The Effects of EE on Neurodevelopment in Brain

The maturation of CNS requires the completion of neuroinduction, proliferation, migration, construction of neural circuits, and myelination, with some existing at a particular time, yet some of which span the entire developmental process. During developmental stage, the neural network of the mammalian brain is susceptible to experience and environment, showing high plasticity. Environmental stimulation is associated to a status of increased brain plasticity (Sale et al., 2014). Animal experimental studies have found that EE can affect the anatomical structure, function, and biochemical changes of CNS, promote the neurogenesis process, and improve the positive behavior of animals. The mechanisms involve brain morphology, cytogenetics, molecular biology, neuroendocrinology, and electrophysiological changes.

### Environmental stimulation and the brain development

#### Impact of prenatal environment on development

External stimuli have a strong impact on the brain development of the fetus. The changes in brain plasticity induced by the stimulation of the external environment do not entirely depend on the direct sensory experience of the developing subject. The embryos or fetuses affected by the intrauterine environment are the first to respond to such changes in organogenesis. The nature of the intrauterine environment depends largely on the prenatal environment experienced by the mother.

Prenatal exposure to adverse environments, such as maternal stress, toxic stimulation, viral infection, and substance abuse, can damage normal brain development. Similarly, the prenatal environment determines susceptibility to pathological conditions in later life. Exposure to injuries that cause adverse conditions *in utero* may negatively affect the development of target organs, destroy the internal microenvironment and enhance the risk of neuropsychiatric disorders in adulthood, including depression, autism and schizophrenia. The biological correlation and mediators between stress and offspring development are mainly related to the hyperactivation of hypothalamic-pituitary-adrenal axis (Gunnar and Quevedo, 2007; Emack and Matthews, 2011), the connection of amygdala and frontotemporal cortex in brain function and microstructure (Favaro et al., 2015), neuronal migration disorders (Hwang et al., 2019), and epigenetic changes (Kundakovic and Jaric, 2017). Numerous animal studies have shown that long-lasting epigenetic changes caused by prenatal stress involve gene encoding BNDF, the corticotropin-releasing factor (Crf), and the glucocorticoid receptor (Nr3c1), which is associated with the HPA axis (Mueller and Bale, 2008; Dong et al., 2015). Recent clinical studies have found that prenatal maternal anxiety predicts accelerated epigenetic age in children in the experimental cohort and reveals increased sensitivity in men rather than woman in regared to the fetal origins of epigenetic age acceleration (Mcgill et al., 2022).

In contrast to adverse stimulation during pregnancy, prenatal positive environment intervention creates favorable conditions for neural development of the embryo and newborn. It is noteworthy that the development of the visual system is sensitive to prenatal EE stimulation. Mice reared in EE showed early eye opening and precocious vision development, which was closely associated with significantly increased precocious cAMP response element-mediated gene expression and higher levels of BDNF and GAD65/67 expression (Cancedda et al., 2004; Sale et al., 2004). Prenatal EE accelerates the migration of neural precursor cells in the retinal ganglion cell layer of the fetus and the natural death of ganglion cells, which depends on the increased insulin-like growth factor-I (IGF-1) induced by enrichment to promote the transfer of nutrients from the placenta to the fetus (Sale et al., 2007). In modern society, pregnant women are extremely vulnerable to active or passive exposure to mobile phone radiation, which will lead to behavioral difficulties in their offspring to varying degrees. Prenatal EE intervention successfully reversed cognitive impairment in elderly offspring caused by long-term radiation exposure during pregnancy through upgrading the synaptic number and the expression of synaptophysin (SYN), postsynaptic density-95 (PSD-95), and BNDF (Hong et al., 2020).

Given that voluntary physical activity is an essential component of EE, we separately analyzed the positive effects of a prenatal exercise intervention on neurodevelopment. More and more studies have shown that reasonable physical exercise during pregnancy is not only conducive to reducing the incidence of gestational diabetes and hypertension and the percentage of body fat in pregnant women but also conducive to promoting fetal development reflected in the increase of placental survival rate and placental hormone secretion (Hopkins et al., 2011; Rosa et al., 2011; Davies and Artal, 2019; Chae et al., 2021). Treadmill training in pregnant rats significantly increased cell proliferation, BNDF, and apoptosis inhibition protein expression in the hippocampus of adolescent and adult progeny rats (Kim et al., 2021), which may explain the high spatial learning ability in neonatal rat pup on the premise of voluntary exercise of maternal rats (Parnpiansil et al., 2003). A meta-analysis of the impact of prenatal exercise on neonatal and child outcomes showed that maternal exercise was associated with a reduced risk of macrosomia, not with neonatal complications or adverse child outcomes (Davenport et al., 2018). In addition to the changes in plasticity of the brain, maternal exercise-induced secretion of Apelin in the placental circulation may provide long-term metabolic benefits to offspring (Son et al., 2022; Wang and Zhu, 2022). These results manifest that prenatal exercise may promote positive development in neonates but may not be effective in inhibiting the occurrence of abnormal diseases, such as those caused by premature birth.

#### Impact of pre-weaning environment on development

The critical period, also known as the sensitive period, is a specific window period in the early postnatal period. During this time window, the development of the brain is susceptible to external stimuli. The development and maturation of the functional characteristics of the brain and the formation of neural circuits are strongly dependent on its own experience and the influence of the external environment (Sengpiel, 2007). The value of stimulation of postpartum mothers to their offspring is equally worthy of attention as the comfortable living environment and psychological state during pregnancy. In mammals, new mothers respond to infants’ unique sounds, touches, and temperatures with attentive responses such as licking and grooming. Infants also enter lactation mode, showing sensitivity to the mother’s basic characteristics (Fleming et al., 1999; Pozzi et al., 2021). Maternal behavior is one of the crucial sources for pre-weaning rodents to obtain experience to promote neural development and neural circuit maturation during the critical period.

One of the most pronounced effects of maternal care is the regulation of stress responses in adult offspring. The body’s ability to cope with stress depends on the programmed changes of the HPA axis in the neuroendocrine system to promote the release of glucocorticoids that respond to early stress stimuli through multiple intracellular sites and mechanisms. Glucocorticoid receptors (GR) play a central negative feedback regulation role on the HPA axis by changing its expression level in hippocampus. The reduction of the intensity of the stress response at the behavioral and endocrine levels and the improvement in cognitive function in adulthood were found in pups of mice and rats which received a brief (3–15 min) maternal separation (Meaney et al., 1996). Another interesting explanation for this result is that pups that were briefly separated from their mothers will receive higher levels of maternal care exhibiting fortified behavior of licking/grooming and arched back nursing, which suggests that the effect of early environmental intervention on pre-weaning pups is mediated by maternal behavior (Liu et al., 2000). However, prolonged (3–6 h) daily mother-pup separation produced the opposite effect to short-term separation, with the pups increasing their response to stress factors in adulthood (van Oers et al., 1998). In addition, weight loss, delayed fur growth and eyes opening, and defects in forelimb grasping of pups were found in the model of maternal deprivation recently (Yang et al., 2021). The harmful effects of early maternal isolation on optimal growth and neurobehavioral development of offspring are not only related to the glucocorticoids and secretory signaling pathways described above, but also involve BNDF synthesis disorders (Ströher et al., 2020) as well as the regulation of epigenetics (Borba et al., 2021).

The detction of impairment of memory and cognitive flexibility elicited by maternal deprivation can be avoided by EE intervention prompts that early EE may accelerate the formation of delicate neural circuits (Menezes et al., 2020; Joushi et al., 2021). The fact that pre-weaning pups are largely unable to engage in voluntary physical activity has led to limitations in EE-related studies focusing on early intervention. But existing researches are enough to prove the positive role that early EE plays in neurodevelopment in which dendrite branches (Venable et al., 1989), neuronal differentiation ability (Pascual and Figueroa, 1996), exploration activities, learning and memory ability (Lu et al., 2017), and inhibitory to excitatory balance (Feng et al., 2021) in the hippocampus are improved observably. Especially in the visual system, early EE can not only promote the development of the visual cortex but also accelerate the development of the retina in terms of substantially no plasticity. The mechanism involved is inseparable from the secretion of neurotrophic factors BDNF and IGF-1 (Landi et al., 2007a, b, Landi et al., 2009).

Without considering the element of voluntary physical activity, we continue to explore reliable evidence that other aspects of EE contribute to the neurodevelopment of pre-weaning pups. On the one hand, in a rich housing environment, mouse pups are more receptive to sensory stimuli, including maternal or non-maternal licking and physical contact such as beard stimulation. Massage that mimics maternal care accelerated the development of the rodent brain and the maturation of visual function (Guzzetta et al., 2009) and promoted weight gain in human premature and low-birth-weight infants (Vickers et al., 2004). Furthermore, the discovery that oxytocin enhanced the motor performance of pre-weaning mice associated with beard stimulation (Tabbaa and Hammock, 2020) also demonstrates that tactile sensory stimulation is an indispensable element for the positive effects of early EE on neurodevelopment. On the other hand, studies have highlighted the importance of social contact during pregnancy and breastfeeding for optimal offspring development in terms of behavior and age. The emotional and cognitive benefits offered by EE are primarily dependent on social interaction. Common nesting (CN) is a model of social enrichment in mice during pregnancy and early lactation. In the CN model, multiple mothers raise more pups, meaning that pups are more exposed to diverse social stimuli. Compared to standard reared, the CN offspring in adulthood showed more social interactivity in task activities, less anxious behavior in the open domain, and more escape attempts in the forced swimming task (Curley et al., 2009; Martinez et al., 2015). Therefore, early EE was mainly focused on physical exercise in pregnancy and more targeted sensory and social stimulation in lactation to promote offspring neural development and neural circuit construction.

#### Impact of environment on development during “adolescence”

Adolescence is the primary postpartum neurodevelopmental period of adult social and cognitive skills maturation, as well as a period of high neuroplasticity of brain development. Rats mature approximately 30 times faster than humans (Quinn, 2005), and the definition of adolescence in animals is based on actual age rather than adolescence, which is roughly divided into early adolescence (P21–34), mid-adolescence (35–46), and late adolescence (47–59; Holder and Blaustein, 2014). The effects of EE on brain plasticity and behavior in adolescent rodents were more significant compared with those before weaning due to the more complex neural circuits and the approaching maturity of the nervous system. In terms of performance of selective and auditory sustained attention, mild adolescent exposure to EE maximizes significant and long-term effects compared to other developmental trajectories such as prenatal or adulthood (Korkhin et al., 2020). EE interventions during adolescence have been used to neutralize the adverse neurodevelopmental hazards of stress during different periods. Postweaned EE can reverse learning and memory impairment in prenatal stress offspring, involving mechanisms associated with lowering plasma corticosterone and increasing the expression of hippocampal IGF-2 and cytoskeletal proteins (Guan et al., 2021). Analogously, spatial learning and memory impairment due to maternal separation before weaning is terminated by enhanced TrkB/BDNF signaling induced by adolescent exposure to EE (Mohammadian et al., 2019; Cordier et al., 2021). Interestingly, new objective recognition of memory and anxiety-like behaviors effects of EE exposure in adolescence appear sex-dependent (Sakhaie et al., 2020).

In addition, we analyzed the contributions of physical exercise and EE to neurodevelopment during adolescence (Table 1). Both pubertal exercise and EE ameliorated deficiencies in water maze learning, hippocampal LTP, and BDNF levels elicited by prenatal morphine exposure (Ahmadalipour et al., 2018). In terms of anxiolytic and activity levels, EE and exercise also had comparable effects on CD-1 mice (Aujnarain et al., 2018). Exercise alone can lead to some impacts that appear to be similar to the effects of EE. Still, detailed analysis will reveal how different these seemingly identical effects actually are, such as the the expression of some protein levels in the hippocampus. In the studies of hippocampal plasticity, it was found that IGF-1 responded positively to EE rather than exercise training. The opposite result was obtained in terms of declining corticosterone and parvalbumin expression. Physical activity was superior to EE for an increase in the number of cells in the CA1 (Serra et al., 2020; Rostami et al., 2021). In addition to the changes in the hippocampus, EE was able to regulate leptin sensitivity in young mice and leave metabolic and synaptic imprints to prevent obesity, the mechanism of which involves an increase in activation of STAT3 and a transfer of the excitation/inhibition balance toward the form er in α-melanocyte-stimulating hormone (α-MSH) neurons and toward the latter in agouti-related peptide (AgRP)/Neuropeptide Y (NPY) neurons. However, the above changes produced by physical exercise are not significant as EE and do not involve an effect on synaptic connections (Mainardi et al., 2010). The above results suggest that physical exercise, one of the components of EE, is not in any way weaker than or equal to EE in its impact on neurodevelopment and maturation.

**Table 1:** Effects of physical activity and EE on brain development.

**Period**	**Intervention**	**Strategy**	**Mechanisms**	**Effects**	**References**
Pregnancy	EE	44 × 62 × 28 cm	Increasing expression of precocious cAMP response element-mediated gene, BDNF and GAD65/67	An earlier eye opening, a precocious development of visual acuity, and an accelerated decline of white matter-induced long-term potentiation	Cancedda et al. (2004) and Sale et al. (2004)
	EE	100 × 50 × 82 cm	Increasing expression of IGF-1	Accelerating the migration of neural progenitor cells in the fetal retinal ganglion cell layer and the natural death of ganglion cells and promoting the transfer of nutrients from the placenta to the fetus	Sale et al. (2007)
	EE	60 × 45 × 76 cm	Increasing expression of SYN, PSD-95, an BNDF	Reversing cognitive impairment in elderly offspring caused by long-term radiation exposure	Hong et al. (2020)
	Voluntary exercise	Squat exercise and tower climbing	No found	Heavier and longer mid-uterine horn fetuses and greater placental weight	Rosa et al. (2011)
	Treadmill exercise	At a slope of 0°at 2 m/min for the first 5 min, 5 m/min for the next 5 min, and 8 m/min for the last 20 min	Increasing expression of Bcl-2, BDNF, and TrkB Inhibiting expression of Bax	Promoting hippocampal cell proliferation to improve spatial learning	Kim et al. (2021)
	Treadmill exercise	The intensity of activity in the early, middle and late stages of the embryo was 40% VO_2_max, 65% VO_2_max, and 50% VO_2_max	Activation of apelin-AMPK signaling	Protecting offspring from diet-induced obesity and metabolic disorders	Son et al. (2022)
Pre-weaning	EE	70 × 70 × 40 cm	No found	Increasing basal dendrite branches of pyramidal neurons in occipital cortex	Venable et al. (1989)
	EE	40 × 70 × 100 cm	No found	Promoting differentiation of pyramidal neurons in motor and visual cortex II and III	Pascual and Figueroa (1996)
	EE	62 × 50 × 40 cm	Increasing field excitatory postsynaptic potentials and activation of ERK signaling	Enhancing hippocampal-dependent learning and memory function	Lu et al. (2017)
	EE	60 × 45 × 20 cm	Maintaining the inhibitory to excitatory balance in the hippocampus	Promoting experience-dependent inhibitory plasticity	Feng et al. (2021)
	EE	60 × 50 × 80 cm	Increasing expression of IGF-1 and BDNF	Speeding up the development of the retina	Landi et al. (2007a)
Adolescence	EE	90 × 50 × 60 cm	Increasing expression of IGF-2 and activity-regulated cytoskeletal-associated protein	Repairing learning and memory impairment in offspring of prenatal stress	Guan et al. (2021)
	EE	90 × 60 × 75 cm	Increasing expression of TrkB and BDNF	Improving the effects of maternal separation on spatial learning and memory	Cordier et al. (2021)
	Treadmill exercise	Running at a speed of 2 m/min for the first 5 min, 5 m/min for the next 5 min and then at a speed of 8 m/min for the last 20 min, at a 0° inclination	Enhancing LTP and expression of BDNF	Reversal of spatial learning impairment induced by prenatal morphine exposure	Ahmadalipour et al. (2018)
	EE	100 × 100 × 50 cm			
	Treadmill exercise	Running time and speed started at 8 m/min for 5 min and gradually increased over this period, reaching a maximum of 18 m/min for 60 min	Increasing the total number of parvalbumin cells of subiculumand CA2/3 region (EX>EE)	Promoting early brain development	Aujnarain et al. (2018)
	EE	60 × 30 × 49 cm			
	Combined exercise training	Aerobic and resistance training was performed on even and odd days	Increasing expression of IGF-1 Inhibiting expression of corticosterone and the cell numbers of the hippocampus (EE>EX)	Promoting the downstream plasticity effects on the hippocampus	Rostami et al. (2021)
	EE	40 × 60 × 90 cm			
	EE	44 × 62 × 28 cm	Reducing leptin release and increasing leptin sensitivity and the activation of STAT3 pathway (EE>EX)	Improving glucose tolerance, feeding behavior, and leptin sensitivity to prevent obesity	Mainardi et al. (2010)
	Physical exercise	Free access to a running wheel	Ehancing synaptic connections (Only in EE)		
Adutlhood	EE	86 × 76 cm (No found in height)	No found	Increasing the volume of the hippocampal structure and sensorimotor cortex	Scholz et al. (2015)
	EE	46.2 × 40.3 × 40.4 cm	Promoting the survival of the neuronal precursors in the hippocampal DG	Promoting neurogenesis in the hippocampus	van Praag et al. (1999)
	Physical exercise	Running wheel for voluntary	Accelerating cell proliferation		
	EE	45.5 × 21.5 × 12 inches	Activating FGFR signaling	Promoting neurogenesis in the hippocampus	Grońska-Pęski et al. (2021)
	Physical exercise	Two running wheel for voluntary			
	EE	85 × 75 × 40 cm	Increasing oligodendrocyte-specific RNAs expression	Adding more new axons with sheaths and improving motor coordination	Goldstein et al. (2021)
	EE	24 × 20 × 46 cm	More oligodendrocyte progenitor cells proliferation and oligodendrocyte mature cells in the sensorimotor cortex	Accelerating axon development	Keiner et al. (2017)
	EE	82 × 61 × 45 cm	Increasing spinophilin mRNA expression	Increasing dendritic sprouting and number of dendritic spines	Hu et al. (2010)
	EE	36 × 23 × 18 cm	Increasing synaptophysin and PSD-95 expression	Improving synaptogenesis	Nithianantharajah et al. (2004)
Aging	EE	120 × 100 × 60 cm	Enhacing the extracellular concentrations of glutamate and GABA in the hippocampus	Promoting neurogenesis in the hippocampus	Segovia et al. (2006)
	EE	44 × 62 × 28 cm	Reducing the inflammatory chemokine ccl11/eotaxin-1 expression	Improving learning and memory function in elderly mice	Scabia et al. (2021)

Abbreviations: IGF-1, insulin-like growth factors -1; BDNF, brain-derived neurotrophic factor; IGF-2, insulin-like growth factors -2; TrkB, Tyrosine Kinase receptor B; ERK, extracellular regulated protein kinases; LTP, Long-term potentiation; AMPK, AMP-activated protein kinase; Bcl-2, B-cell lymphoma-2; Bax, BCL2-Associated X; DG, dentate gyrus; FGFR, fibroblast growth factor receptor; PSD-95, postsynaptic density-95; EX>EE, the effect of exercise is more significant than EE; EE>EX, the effect of EE is more significant than exercise.

### Neural consequences of EE

#### Brain volume

The early discovery that wild rats and mice living in EE not only have increased active exploration behavior but also possess more outstanding learning and memory abilities and problem-solving abilities than animals in standard rearing cages attract researchers to study the anatomical changes caused by EE Compared with the normal group, male rats exposed to EE for 30 days showed increased dry and wet brain weight and cortical thickness, especially occipital somesthetic and dorsal cortex (Bennett et al., 1969). Subsequent, more detailed study of EE’s effects on the cerebral cortex suggested that the changes of cortical thickness were independent of the age of animals, and 30 days instead of a longer time EE had the broader effects on cortex depth (Diamond et al., 1972). This finding may be related to the decline in environmental novelty due to long-term EE. Outside the cortex, EE also aggrandizes the hippocampus, striatum, and midbrain volume (Scholz et al., 2015).

#### Neurogenesis

Changes in the appearance of the brain have also led scientists to explore microscopic structures. The influence of EE on the number of cells in the adult brain dates back to 1964. A study investigating whether EE enhances the number of neurons found only the genesis of neurogliacyte (Altman and Das, 1964). The probable reason for the failure of new neuron observation was that the research focuses on the cortex rather than the hippocampus. Subsequent studies demonstrated that the increased glial cells were mainly derived from astrocytes (Walsh et al., 1969; Kronenberg et al., 2007) and oligodendrocytes (Szeligo and Leblond, 1977), whose cell morphology was also affected by EE. The 1997 study was the first to suggest that EE increased the number of hippocampal nerve cells in adult mice (Kempermann et al., 1997). Larger hippocampal granulosa cell layers and the 15 percent more granule cell neurons in the dentate gyrus (DG) were discovered in mice living in EE Adult neurogenesis is a crucial mechanism for the beneficial effects of positive living, enabling mice in EE to learn more flexibly (Garthe et al., 2016). Only neurons that undergo a complete process of proliferation, migration, differentiation, and survival can be called successful neurogenesis. Voluntary exercise-induced hippocampal neurogenesis was attributed to cell proliferation, and neuronal survival did not differ from the standard group. Conversely, EE promotes the survival of the neuronal precursors in the hippocampal DG but has no significant effect on the proliferation of neural progenitor cells (van Praag et al., 1999). This means that the mechanism by which new cells are produced in the same genetic context depends on different living environments.

Furthermore, the relationship between the effect of EE on neurogenesis and the age of the target is also quite subtle. Based on the study that EE expedites neurogenesis in adult hippocampus, exposure of adult mice aged 10–20 months to EE showed a fivefold increase in hippocampal neurogenesis (Kempermann et al., 2002). Similarly, comparison of neurogenesis in hippocampal DG area of old and young rats shows that the number of newborn cells of old and young rats is significantly higher than that of control rats after EE, even though the number of newborn cells of old rats is markedly less than that of young rats (Segovia et al., 2006). The above experiments indicate that EE can exert a long but not short effect on neuroplasticity across an extensive age range. However, mice that received EE stimulation 7–21 days after birth was detected with significant differences in autonomic movement but no substantial changes in hippocampal nerve cell proliferation at the age of 4 months compared with the control group (Kohl et al., 2002). This suggests that EE is not highly correlated with hippocampal neurogenesis and the maintenance of neuroplasticity. The reason for this result may be due to the small amount of exercise in mice before weaning and the confusion of EE and maternal effect. Given the age of EE’s target and the specific EE strategies, it remains to be seen whether the impact of enrichment on the plasticity of the CNS is maintained over the long term.

At present, the research on the mechanism of EE affecting neurogenesis is still being explored. Early experiments have shown that EE can enhance the growth factors that promote neurogenesis, including brain-derived neurotrophic factor (BDNF; Zorzin et al., 2021), nerve growth factor (NGF; Pham et al., 1999), glial cell-derived neurotrophic factor (GDNF; Young et al., 1999), vascular endothelial cell growth factor (VEGF; Cao et al., 2004). A recent study suggests that EE and exercise can also enhance adult hippocampal neurogenesis through fibroblast growth factor receptor (FGFR), which induces the proliferation of stem and progenitor cells (Grońska-Pęski et al., 2021).

#### Neurite growth and synaptogenesis

In addition to affecting the production of new hippocampal DG neurons, EE also leads to further morphological changes. Axon plays a vital role in the functional connection between neurons and target cells at any stage of life. The formation of CNS complex networks is highly dependent on the degree of axon differentiation during normal development. Early EE intervention extended the axon initial segment of the neocortex to modulate cell excitability (Nozari et al., 2017) and lengthened the axon terminals in the absence of any nerve damage (Fernández-Montoya et al., 2018). Semaphorin-3a of perineuronal nets, a kind of axon growth inhibition factor, was significantly reduced after EE (De Winter et al., 2016). EE for 30 days did not initially increase oligodendrocytes and myelin expression in the white matter. Follow-up studies were surprised to find that 60 days of EE enhanced the g/ratio and more new myelinated axons in 90 days of EE, which suggests that the effects of EE on axons begin with the thickening of myelin fibers followed by an increase in the number of internodes (Goldstein et al., 2021). In addition, more oligodendrocyte progenitor cells proliferation and oligodendrocyte mature cells were detected in the sensorimotor cortex at 10 and 42 days of EE intervention, respectively (Keiner et al., 2017), which indicats EE will accelerate axon development. Unfortunately, there are relatively few studies on EE and positive growth and correct guidance of axons under normal development.

Dendrites, one to more protrusions, emanated from the cell bodies, are responsible for receiving stimuli and transmitting nerve impulses to the cell body of other nuerons. The length, number, branches, and other developmental conditions of dendrites can affect the reception of signals and thus influence the formation of neural circuits. Earlier studies have shown that EE leads to the development of higher-order dendritic branches and denser dendritic spines in vertebral neurons of the occipital (Greenough and Volkmar, 1973), frontotemporal (Greenough et al., 1973), and parietal lobes (Leggio et al., 2005). However, the molecular mechanisms underlying changes in dendritic structural plasticity have rarely been explored. Spinophilin is highly enriched in dendritic spines and is involved in spinal morphology and synaptic plasticity. One week after EE, the density of spinophilin immunoreaction in the hippocampus and cortex augmented instantaneously. In the following 3 weeks of EE intervention, spinophilin mRNA continued to grow (Hu X.-L. et al., 2010).

It is well known that the positive response of dendritic morphology and associated synaptic proteins are strong evidence that EE improves synaptogenesis. EE rats showed a significant increase in SYN, synapsin I, and PSD-95 protein expression (Nithianantharajah et al., 2004; Birch et al., 2013). Interestingly, the distinction between EE physical and social elements was found to have different effects on synaptogenesis. The density of dendritic spines increased in the DG of the social group and the CA1 of the activating group (Gabriel et al., 2020). The reasons for this need to be further explored according to the anatomical characteristics of the hippocampus and different environmental factors. Furthermore, BDNF plays an essential role in EE-induced morphological plasticity of dendritic spines and hippocampal synaptogenesis (von Bohlen und Halbach and von Bohlen und Halbach, 2018). The exact mechanism underlying these effects remains uncertain. The research found that the kinesin superfamily motor protein 1A (KIF1A) was specifically upregulated in the hippocampal region of EE mice. Excitingly, the level of KIF1A and KIF1A mediated cargo transport was positively correlated with BDNF expression. Subsequent studies demonstrated that EE could not induce induced hippocampal synaptogenesis and learning enhancement *via* BDNF in KIFIA knockout mice (Kondo et al., 2012). The result may be applied to explain the mechanism of motion elements of EE in the future.

## The Effects of EE on CNS Disorders

In addition to being widely used in the study of normal animal models, EE is also widely used in the study of various neurological/psychiatric disease models, such as AD, PD, HD, stroke, epilepsy, down syndrome, drug addiction, depression, schizophrenia, attention deficit, and hyperactivity disorder, etc. Studies have shown that EE has beneficial effects on these diseases, and people have even explored the potential of EE as a means of treatment and rehabilitation for these diseases (Kotloski and Sutula, 2015). Here we focus on the neural repair mechanisms of EE in the CNS, especially in stroke.

### EE and cerebral ischemia

Stroke is the second leading cause of death and disability worldwide. Cerebral ischemia caused by arterial occlusion is a common type of stroke. The death of neurons in the ischemic core result in irreversible neurodevelopmental damage. Although some molecular mechanisms related to the pathogenesis of cerebral ischemia have been explored in animal experiments, there is still a lack of comprehensive understanding of its pathological process. Due to the current treatment of patients in the subacute stage and acute stage being mainly single drug treatment, more and more scientific research has turned to nonpharmacologic strategies, in which the study of EE on the repair of neurodevelopmental disorders after cerebral ischemia has been carried out in multiple dimensions.

At the level of brain structure, the results of most studies on whether EE can reduce the volume of cerebral infarction are positive (Gonçalves et al., 2018; Deng et al., 2021; Liu et al., 2021). At the same time, the EE group rats showed fewer neurological deficits after ischemia, which hints that EE may save the death of ischemic penumbra neurons. Of course, EE had little effect on the infarct in a subset of ischemic studies (Dahlqvist et al., 1999; Yu et al., 2013a). Surprisingly, premature exercise or administration of EE even enlarged infarct size (Risedal et al., 1999). The results of meta-analysis also support the above view. The reason may be that in the early stage of cerebral infarction, the fear caused by the need for experimental animals to adapt to the new environment results in delayed tissue injury and increased infarct volume (Janssen et al., 2010). In other words, since most EE interventions are performed 24 h after ischemia, changes in the new environment compared with the initial living environment, including surrounding scene and social interaction may increase the possibility of late tissue loss. The difference of EE on cerebral infarction volume outcome may also be related to various EE settings. Therefore, the relationship between EE and infarct volume needs to be discussed systematically, and the factors such as age, gender, and strain of experimental animals need to be considered.

As with normal neural development, EE also increases neural stem cell and progenitor cell proliferation and neurogenesis of the subventricular zone (Komitova et al., 2005) and hippocampus (Matsumori et al., 2006) after cerebral ischemia. The mechanism of EE promoting neurogenesis after cerebral ischemia is largely related to gliogenesis, especially in astrocytes. On the one hand, EE can comprehensively improve the neurological function of stroke rats through BDNF released by proliferating astrocytes (Chen et al., 2017). On the other hand, EE promotes cytoplasmic to nuclear transfer of nuclear factor κB (NF-κB)/p65 in activated astrocytes, which enhances gene expression of interleukin 17A to facilitate neurogenesis directly (Zhang et al., 2018). It is widely accepted that rehabilitation after stroke requires the cooperation of nerve and vascular tissues. Effective neurovascular reconstruction after ischemia plays a crucial role in the repair of subsequent developmental injury. EE rapidly promotes angiogenesis through different mechanisms to improve physical and cognitive recovery in animal models of ischemic stroke. A significant increase in angiogenesis specific marker CD31 was observed in the cortical area around infarction after EE, and the neurological function was positively correlated with angiogenesis (Yu et al., 2014). When sub-components were isolated to dissect their contribution to the total effect of EE by rearing animals either by stimulating physical exercise, or under social enrichment alone, the results showed that complete EE had a more significant benefit than single sub-components on microvessel density, as manifested by elevated expression of angiopoietin 1, VEGF, and CD31 (Zhang et al., 2021). The mechanism underlying angiogenesis may be mediated by hepatocyte growth factor, a blood-derived factor, which was elevated by EE in cortex and serum after middle cerebral artery occlusion (MCAO). The statement stems from the finding that survival and proliferation of endothelial cells were significantly improved when co-cultured with serum and plasma derived from EE after ischemia (Xie et al., 2019). In addition, magnetic resonance angiography showed selective amplification of basilar, internal carotid, and middle cerebral artery signals after MCAO *via* a three-stage EE paradigm designed according to different stages of cerebral ischemia, which was regulated partially by phosphatidylinositol 3-kinase (PI3K)/protein kinase B (AKT)/glycogen synthase kinase-3 (GSK-3)/β-catenin and the axon guidance molecules (Zhan et al., 2020).

At the molecular level, the repair of damaged brain tissue after cerebral ischemia is dependent on the high release of endogenous growth factors. EE mainly enhances BNDF (Shono et al., 2011; Yu et al., 2020), VEGF (Wu et al., 2018), and NGF (Dahlqvist et al., 2003) to induce neurogenesis, angiogenesis, and synaptogenesis after reperfusion to achieve recovery of physical and cognitive functions. At the functional level, post-stroke cognitive impairment (PSCI) is a major obstacle on the road to neural restoration. We have systematically summarized the recovery mechanism of PSCI after EE paradigms, including cell death, synaptic plasticity, cholinergic circuit, and cerebral blood flow (Yuan et al., 2021). It should be added here that, in addition to classic apoptosis, the latest research shows that EE also alleviates pyroptosis by inhibiting NF-κB/p65 to ameliorate neurological deficits (Liu et al., 2021). Similarly, it is necessary to explore the relationship between other forms of cell death and EE promoting recovery in the future, such as ferroptosis, which occurs largely after cerebral ischemia (She et al., 2020). Brain plasticity is not only the principle of normal brain development, but also plays an irreplaceable role in nerve repair after injury besides spontaneous repair. A recent study has indicated that EE can increase the expression of growth-associated protein (GAP-43) and SYN in bilateral cerebral hemispheres. Interestingly, the incremental expression of synapse-associated proteins on the contralateral side of injury has a higher correlation with cognitive function compared with the ipsilateral side (Wang et al., 2019). In addition to the cholinergic circuit (Wang et al., 2016; Yuan et al., 2020), the upregulation of GABAergic and glutamatergic systems under the effect of EE in the contralateral hippocampus is also committed to the remission of PSCI (Wang et al., 2020), which suggests that EE recovery of PSCI is no longer limited to the damaged hemisphere at least in terms of synaptic plasticity and neurotransmitter system.

### Other neurobiological disorders

In addition to cerebrovascular accident, the structural and functional impairment caused by neurodegenerative diseases can be repaired effectively. Studies have found that EE exposure can reduce the probability of hippocampal nerve injury in AD animals, enhance synaptic plasticity, and reduce hyperphosphorylated tau protein and cortical amyloid β-protein in the hippocampus. The abundance level of amyloid improves the decline of memory function (Hu Y.-S. et al., 2010; Valero et al., 2011; Salmin et al., 2017). A study based on resting fMRI found that EE exposure enhanced the functional connection between hippocampal CA1 and cortex of AD transgenic animals (Little et al., 2012). EE exposure can alos reduce the loss of dopaminergic neurons in substantia nigra compacta of PD animals, reduce the expression of dopamine transporter expression (DAT), increase the level of BDNF and improve motor dysfunction (Goldberg et al., 2011; Fischer, 2016). EE exposure can also restore the level of BDNF in HD animals, improve motor function, restore hippocampal neurogenesis, and alleviate the atrophy of cerebral cortex volume (Nithianantharajah and Hannan, 2006; Fischer, 2016). Three weeks of EE exposure can enhance the resistance of rats to seizures, reduce hippocampal cell apoptosis, promote hippocampal neurogenesis, increase the expression of GDNF and BDNF, increase animal exploration activities and improve spatial learning ability. Even in the absence of neurodegenerative diseases, a decline in cognitive abilities is accompanied by brain aging. Recent studies have demonstrated that EE can improve learning and memory function in elderly mice by reducing pro-inflammatory chemokines ccl11/eotaxin-1, a marker of aging (Scabia et al., 2021).

Moreover, EE exposure can also reduce the strengthening and seeking of psychostimulants (such as cocaine, amphetamine, morphine, nicotine, etc.) and reduce the risk of relapse. These behavioral changes caused by EE are related to the reduction of the functional activities of some brain regions involved in drug seeking and relapse, such as nucleus accumbens, dorsal striatum, amygdala, ventral tegmental area substantia nigra, prefrontal cortex, and cingulate cortex (Solinas et al., 2008, 2010; Thiel et al., 2010; Chauvet et al., 2011; Gill et al., 2014; Sikora et al., 2018) and are related to the reduction of the drug-induced DAT in the striatum and prefrontal cortex, dopamine uptake and the rate-limiting enzyme of dopamine synthesis-tyrosine hydroxylase (Zakharova et al., 2009; Läck et al., 2010; Wooters et al., 2011). In animal models of anxiety and depression, EE also has similar anti-anxiety and anti-depression effects. For example, animals with EE showed lower pleasure deficiency-like behavior in the sugar water preference test, less social withdrawal in the social interaction test and less immobility in the forced swimming test (Zhang et al., 2014).

## Challenges and Expectations Facing EE

### Effect weight of stimulus in EE

EE is mainly composed of novel sensory stimulation, sports, and social interaction. A study of perinatal hypoxic-related white matter damage in the brain suggests that a single running wheel, a single novel sensory stimulus, and social interaction have little behavioral and cellular molecular impact on the repair of white matter damage. Only complete EE can have the best effect on neural development and repair, which is basically consistent with the results of the EE element separation experiment mentioned above (Forbes et al., 2020). It shows that the impact of a any factor of EE cannot be isolated easily, and its influence is the interaction between various essential elements nor the result of any single aspect. However, it is unknown whether different factors play different roles in the effects of EE and the weight of the importance of the three factors. Again, this needs to be demonstrated by more rigorous EE element separation experiments.

### Window period and duration of EE

Finding the optimal intervention window is critical for CNS recovery. Studies have shown that delayed, late, and prolonged EE do not cause changes in brain plasticity and function in neonatal hypoxia models (Forbes et al., 2020). Early EE intervention for a week only resulted in differential changes at the cellular level, such as promoting oligodendrocyte differentiation and the production of more oligodendrocyte lineage cells. EE intervention lasting 30 days had positive behavioral and cellular molecular effects. Furthermore, EE has made a breakthrough in the research on promoting the visual system’s development to explore the intervention window. EE accelerates brain development by acting on the opening and closure of the critical period, as well as being able to reopen the critical period in the adult animal (Morishita and Hensch, 2008). The underlying molecular mechanisms include enhanced serotonin release, decreased inhibitory/excitatory balance, and increased expression of BDNF and IGF-1 (Sale et al., 2014). Reopening the sensitive stage of adult brain plasticity may eventually lead to the restoration of visual function in adult animals with amblyopia at the functional level while improving the plasticity of the visual cortex. Spatially, most EE is performed after the onset of the disease. Encouragingly, EE preconditioning was also associated with injury repair, as demonstrated in models of acute retinal ischemia (González Fleitas et al., 2019) and cerebral ischemia (Yu et al., 2013b). The mechanism of EE preconditioning may be partly related to the reduction of oxidative stress and inflammatory response.

In addition, in terms of time, current animal model studies generally adopt long-term EE exposure, but there are few studies on the impact of a week or a few days of EE expose-related brain. In fact, 24 h of EE exposure was sufficient to improve target recognition memory and was maintained after a more extended period of EE exposure (Kobayashi et al., 2002; Leger et al., 2015). Even dozens of minutes and hours of novel EE exploration can lead to increased activity in multiple brain regions, including the motor cortex, prefrontal cortex, striatum, and hippocampus, etc. (Ali et al., 2009; Rinaldi et al., 2010). A recent study on manganese-enhanced magnetic resonance imaging *in vivo* found that subacute EE exposure at 7 days resulted in volume changes in multiple brain regions. At the same time, EE-related functional changes are not always accompanied by structural changes, and functional changes do not always accompany structural changes. Still, there is a robust structural connection between brain regions with increased volume and brain regions with increased functional activity (Li et al., 2020). Therefore, subsequent studies on EE should appropriately focus on preconditioning and short-term intervention in order to explore the mechanism of EE from a broader dimension.

### Clinical transformation of EE

The classical theory holds that individual phenotype is determined by the interaction of genetic background and environment (Rogers et al., 2019). EE consists of shared environment (such as urban transportation, urban buildings, work units, etc.) and non-shared environment (such as family residence, family background, physical characteristics, etc.). In the same shared environment, individual phenotype differences depend on genetic background and non-shared environment. Individual responses to environmental stimuli are different, which may indicate that the individual steady state has varying degrees of influence on brain plasticity. Recent studies have shown that early EE can lead to persistent individualized changes in behavior, brain plasticity, and epigenetics, even under the condition of controlling genetic background and shared environment (Kempermann, 2019; Zocher et al., 2020). Ethology and epigenetics may be one of the most meaningful topics in future EE exploration.

However, in terms of clinical transformation, it is easier to increase shared environment and reduce non-shared environment as much as possible for inpatients, such as the rehabilitation treatment after stroke in the hospital. However, for out-of-hospital patients, not only the shared environment is variable, but also the non-shared environment is diverse, which is the difficulty of EE transformation. In addition, there are many technical constraints: (1) how the rearing condition in animal experiments correspond to the mode and environment of treatment in clinical practice, and whether the transformation of standardized cages into standardized housing means that life lacks characteristics; (2) it is difficult to achieve standardized environmental conditions between hospitals or between hospital departments let alone the intervention of patients outside hospitals; (3) there is currently a lack of theoretical knowledge on what kind of environmental integration can promote brain plasticity. For the clinical transformation of EE, we can first clarify the molecular mechanism of EE action in animal models, find the critical molecular substrate of EE effect, and develop drugs simulating the action of EE for this molecule. The study demonstrating that reduction of intracortical inhibition can partly restore ocular dominance plasticity in adult rats suggests a possible strategy to mimic the effects of EE (Harauzov et al., 2010). This would lay the ground for reviewing the possible strategies used to reproduce the effects of EE (e.g., by using fluoxetine; Maya Vetencourt et al., 2008) in a more “standardized” manner, with the goal of minimizing the interindividual variations in response to EE. Perhaps elucidating the neural mechanism of EE action and developing devices based on this mechanism to regulate related neural activity is a good choice. Although the road ahead is fraught with difficulties, the future of clinical transformation of EE is still foreseeable.

## Conclusion

At present, both animal experiments and clinical studies have proved that environment-dependent brain plasticity is crucial for normal brain neural development and the repair of neurological disorders. EE not only changes basic brain anatomy, but also modulates electrophysiology, neurotrophic factors, and protruding plasticity. At the same time, the multi-dimensional effect of EE on nerve repair in CNS injury has enabled researchers to see therapeutic approaches that do not rely on drugs. However, there are also some contradictory studies, such as the influence of EE on the size of cerebral infarction. Although the level and dimension of EE research are more and more extensive, it still faces many challenges. In the future, window periods and clinical transformation of EE are urgently needed. At the same time, the research direction of EE on neural development and repair should be carried out in a broader direction, among which the study of individuation and epigenetics is worthy of in-depth exploration.

## Author Contributions

MY and Y-SG designed the structure of the manuscript. X-YS and Z-KG managed the literature searches and analyses. YH wrote the manuscript. XB assisted with the improvement of the manuscript. All authors contributed to the article and approved the submitted version.

## Conflict of Interest

The authors declare that the research was conducted in the absence of any commercial or financial relationships that could be construed as a potential conflict of interest.

## Publisher’s Note

All claims expressed in this article are solely those of the authors and do not necessarily represent those of their affiliated organizations, or those of the publisher, the editors and the reviewers. Any product that may be evaluated in this article, or claim that may be made by its manufacturer, is not guaranteed or endorsed by the publisher.
